# A neural hallmark of auditory implicit learning is altered in older adults

**DOI:** 10.1371/journal.pone.0211468

**Published:** 2019-01-30

**Authors:** Sarah E. Donohue, Steffi Weinhold, Mircea A. Schoenfeld, Rodrigo Quian Quiroga, Jens-Max Hopf

**Affiliations:** 1 Department of Neurology, Otto-von-Guericke University, Magdeburg, Germany; 2 Leibniz Institute for Neurobiology, Magdeburg, Germany; 3 Kliniken Schmieder Heidelberg, Heidelberg, Germany; 4 University of Leicester, Leicester, United Kingdom; University of Zurich, SWITZERLAND

## Abstract

Temporal regularities in the environment are often learned implicitly. In an auditory target-detection paradigm using EEG, Jongsma and colleagues (2006) showed that the neural response to these implicit regularities results in a reduction of the P_3_-N_2_ complex. Here, we utilized the same paradigm, this time in both young and old participants, to determine if this EEG signature of implicit learning was altered with age. Behaviorally, both groups of participants showed similar benefits for the presence of temporal regularity, with faster and more accurate responses given when the auditory targets were presented in a temporally regular vs. random pattern. In the brain, the younger adults showed the expected decrease in amplitude of this complex for regular compared to irregular trials. Older adults, in contrast, showed no difference in the amplitude of the P_3_-N_2_ complex between the irregular and regular condition. These data suggest that, although auditory implicit learning may be behaviorally spared in aging, older adults are not using the same neural substrates as younger adults to achieve this.

## Introduction

Determining the presence of regular patterns in the sensory information we receive is one way in which we can make sense of the world around us. Such patterns can help us predict upcoming events, and indicate when something deviates from expectation, potentially alerting us to danger. Interestingly, regularities can often be learned without conscious awareness of their existence, i.e., learned implicitly (e.g., [1]). How such learning evolves over a lifetime is of much interest, as a decline in this ability is present as part of healthy aging, at least under certain circumstances (see [2] for review).

Implicit learning is often studied using variants of the Serial Response Time task (SRT), which was first developed by Nissen and Bullemer [3]. In their study, Nissen and Bullemer presented subjects with either a repeated sequence or a random sequence of visual stimuli. They observed that participants were faster to respond to the repeated sequence, suggesting that this sequence was learned, and response times were impaired if the repeated sequence was switched to be random. Subsequent work has determined that implicit learning may occur in parallel across spatial and non-spatial domains [4], it can occur in the auditory modality [5], it may depend on both task order and response order to be constant [6], and it can still occur if the participants are not aware of any patterns [7].

The examination of implicit learning effects across a lifespan has revealed mixed results. Studies providing evidence for preserved implicit learning in healthy older subjects have only observed such preservation under specific conditions (e.g., [8–15]). For example, Weiermann and Meier [13] found that older and younger adults showed similar learning effects of sequences, but only when there was explicit knowledge of this learning in the case of older adults. Curran [8] found that older and younger adults had similar implicit learning for simple first-order predictive sequences, but older adults showed impaired learning in more complex sequences. The sparing of implicit learning in aging may also be related to the level of education and verbal ability, with older adults of lower ability showing deficits in implicit learning [16]. Indeed, although implicit learning may be slightly spared in healthy aging, this is not consistently found (see [17]), and it is often the case that some sort of deficit appears when comparing older adults to younger (e.g., [18–21], suggesting that this system is not completely spared from the general decline in memory that occurs in healthy aging.

The aforementioned studies have used behavioral measures to determine the effects of implicit learning, and given that there have been some differences observed between older and younger participants, it is likely that such differences arise through activity in different neural substrates. Simon and colleagues [22], demonstrated that in younger adults there was a shift in implicit learning after training to recruit the caudate instead of the hippocampus, whereas older adults showing impaired behavioral learning and no such shift in neural activity. Even when behavioral performance was similar across groups of older and younger adults, further evidence of differential neural activity comes from Dennis and Cabeza [23], who found that younger adults showed activity for implicit memory in the caudate and putamen, whereas older adults showed more activity in the medio-temporal lobe (MTL). Several recent reviews have suggested that because implicit learning is largely mediated by the striatum, and older adults have shown to have a decline in function of this region, it is likely that other areas such as the MTL play a compensatory role, which is, perhaps, not successful for all types of implicit learning [2,24].

If the implicit learning of patterns can be altered in older adults, then the question arises as to if these differences are visible at the level of single trials, and at what stage of processing these differences emerge. In EEG data from young adults, Jongsma and colleagues [25], found that the P_3_-N_2_ complex is a marker of implicit learning, as it decreased when an auditory target became temporally regular, compared to the target occurring randomly in time. Jongsma and colleagues [25] presented participants with a series of tones, for which half of the time the temporal occurrence of the target tone with respect to background tones was regular for a period of time, and in the other half of the trials the target occurred randomly. Although participants were not aware of the temporal patterns, differences in the responses to these stimuli were present in the P_3_-N_2_ complex. The P_3_-N_2_ –a component that is constructed by subtracting the N_2_ from the P_3_—is known to decrease with target probability and expectancy [26,27]. In other ERP studies of sequence learning, these components are modulated by expectancy, with the violation of contingencies resulting in a larger P_3_ and/or N_2_ [28–30].

In the present study, we implemented the same paradigm used by Jongsma and colleagues [25] to see if this marker of implicit learning differs between older and younger adults. Using a wavelet transformation, which we also implement here, Jongsma and colleagues [25] were able to obtain the amplitude of this component on single trials, thereby tracking learning over the experiment. As a previous ERP study on sequence learning failed to find a modulation of the N_2_b in older adults by sequence deviation [31], we expected to see that the P_3_-N_2_ marker of implicit learning would be altered in older vs. younger subjects. Additionally, we hypothesized that older adults would not show the same behavioral benefits from the auditory sequence repetition that younger adults showed.

## Materials and methods

### Subjects

Twenty-six healthy volunteers were included in this study. All participants were determined to have a normal hearing threshold prior to the start of the experiment. The participants were divided into a young group (N = 13, mean age = 24.5 years, range: 20–30 years, 10 female) and an old group (N = 13, mean age = 71.3 years, range: 63–77 years, 6 female). All 26 subjects were unfamiliar with visual and auditory experiments as well as EEG/MEG measurements. Data from four additional older participants and two additional younger participants were recorded but later excluded due to having an artifact rejection percentage greater than 25%. Participants were paid for their time, and written, informed consent was obtained for each participant. All procedures were approved by the ethics review board at the Otto-von-Guericke University, in Magdeburg, Germany.

### Stimuli and procedure

The stimuli and task were designed to mimic that used by Jongsma et al., 2006. All stimuli consisted of woodblock sounds, each having a duration of 200ms. The background tones had a center frequency of 1.11 kHz, and the target tone had a center frequency of 1.32 kHz. The stimuli were presented via Presentation (Neurobehavioral Systems Inc., Albany, USA). The tones were delivered to the subjects’ ears via plastic tubes (approximately 6m in length), which had a flexible ear-bud-like apparatus at the end that had been individually chosen to fit into the pinna of each subject. All measurements were performed using a sound pressure level of 80 dB.

**Fig 1:**
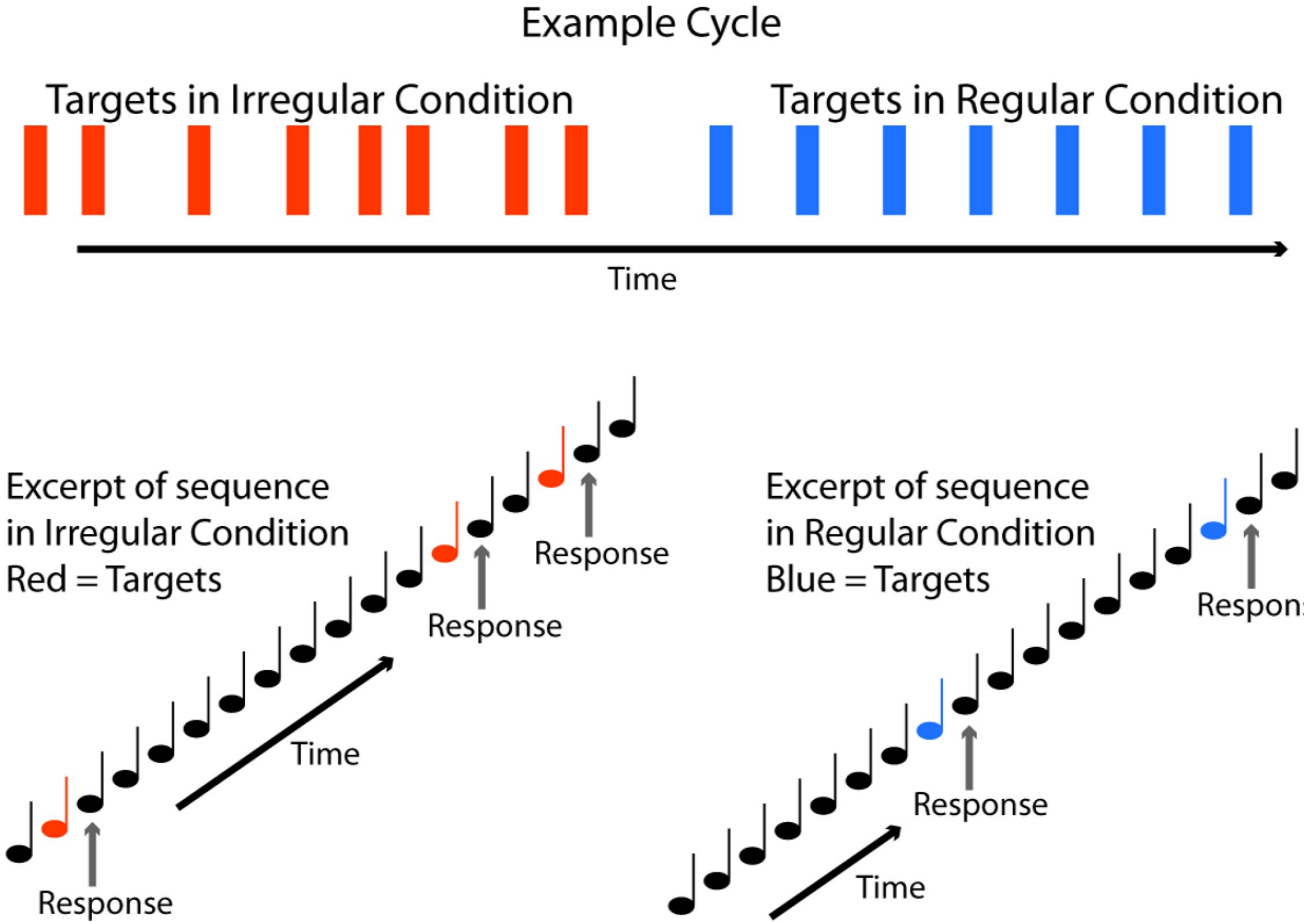
Task. One example cycle is shown (top), with target positions depicted in red for the irregular condition and blue for the regular condition. Background tones (black notes depicted on bottom) were presented every 800 ms between the targets. In the regular condition, eight background tones preceded a target, and this number varied in the irregular condition. Participants responded by pressing a button every time they detected a target tone, but did so after the first background tone following the target.

A continuous train of background tones (ISI = 800ms) was presented to the subjects. The background tones were interspersed with 96 target tones, with a probability of 12.5%. For a detailed schematic of the task, see Fig 1. In total, six consecutive cycles of tones were presented. Each cycle consisted of eight target tones at random positions (irregular condition, IC), followed by eight target tones at fixed positions (regular condition, RC). For both the IC and RC, the overall proportion of targets and background tones was 1:8. In the IC, the target position occurred after either 2–6 or 8–12 background tones, and its position was randomly distributed within the aforementioned constraints. The randomization of presentation in the IC was generated once for the entire experiment (i.e., it was the same across all subjects). In the RC, the targets were always preceded by 7 background tones. Participants were instructed to respond via button press each time a target tone was presented, but to do so after the first background tone following the target tone, so as to eliminate motor-related activity at the time of target presentation. The entire experiment lasted approximately 10 minutes.

### Post-experiment questions

After the experiment, the subjects were asked a couple of questions to determine if they were explicitly aware of the regularity of the target tone. These questions included, “Did you notice anything about the experiment?” and, “Did you notice some kind of regularity, and if so, can you describe it?”

### Recording

The EEG signals were recorded in an electrically shielded chamber using an electrode cap in conjunction with a Synamps amplifier (Neuro Scan, Inc.). EEG data were recorded from 5 scalp channels (Fz, Cz, Pz, Oz, Iz), the left mastoid, and a vertical and horizontal eye channel, with the right mastoid being used as the reference. The impedance of the electrodes was less than 5 kΩ. EEG signals were filtered with a bandpass of DC 50 Hz and digitized with a sampling rate of 254.31 Hz. Subjects were measured sitting in an upright position in a shielded room, lights turned on. To prevent the occurrence of large eye artifacts, participants were instructed to keep their eyes closed during the task.

### Data processing

The data were epoched from +/- 2013ms around the target stimuli (i.e., with the target onset as time 0). Artifact rejection was conducted by removing any epoch with a peak-to-peak amplitude exceeding a threshold of 150 μV. The artifact-free epochs were then exported to ASCII files for further processing in Matlab (MATLAB R2009b, The MathWorks Inc., Natick, MA, 2009). The data were baseline corrected over from -100 to 0 ms. All single-trial ERP responses were de-noised employing an algorithm based on the wavelet transform analysis method [32]. For each ERP component, amplitudes were determined within a particular latency window, which was estimated separately for each subject, based on that subject’s grand average response for all targets. The P_3_ and N_2_ amplitudes were estimated as mean amplitude of +/- 12 ms surrounding the peak values. The P_3_-N_2_ complex was calculated by subtraction of N_2_ from P_3_ amplitude at the midline electrode site PZ, as this was the site of the maximal effect for these components.

### Statistical analyses of EEG data

As mentioned above, six cycles were presented without any breaks between them, with each consisting of eight random targets followed by eight regular targets. We were only interested in the transition from the IC to the RC and not the transition of the RC to the IC, and, to avoid any effects of the latter, we excluded the first two trials of each cycle (i.e., the first two trials of the IC) from the subsequent analysis.

In order to evaluate the overall effect of age and regularity, the amplitudes of the P_3_-N_2_ complex at site PZ were averaged separately for irregular and regular targets, and for young and elderly subjects. The difference in the amplitudes for the irregular minus the regular targets were obtained, and a between-subjects t-test was performed on these differences.

To assess the learning process the trial-by-trial P_3_-N_2_ amplitude changes at PZ were analyzed. If learning had occurred, then one would predict that there would be a decrease in the N_2_-P_3_ complex over the regular trials, indicating the brain’s detection of this regularity. To determine if this were the case, a nonlinear regression analysis employing a gradient-expansion algorithm was performed with F-tests for best-fit computed in IDL (Interactive Data Language version 8.2.1, Exelis Visual Information Solutions, Inc.). If the data could be described with a sigmoid “learning” curve, then it would appear that such learning had occurred. In order to evaluate higher order learning processes, each consecutive cycle was analyzed separately. To test the hypothesis that learning in terms of amplitude reduction occurs more rapidly with each consecutive cycle, an F-test for best fit was performed on the Tn50s (i.e., the point at which half of the amplitude was reached) for all cycles.

### Analysis of behavioral data

Correct responses were defined as responses within a time window of -400 and 400 ms around the first background tone that followed the target stimulus. Reaction times of correct responses (RT) and error rates (ER) were analyzed separately using a repeated measure ANOVA (within-subject factor: condition (irregular, regular); between-subject factor: age (young, old)). All reported ANOVAs were Greenhouse-Geisser corrected.

## Results

### Behavioral data

**Fig 2:**
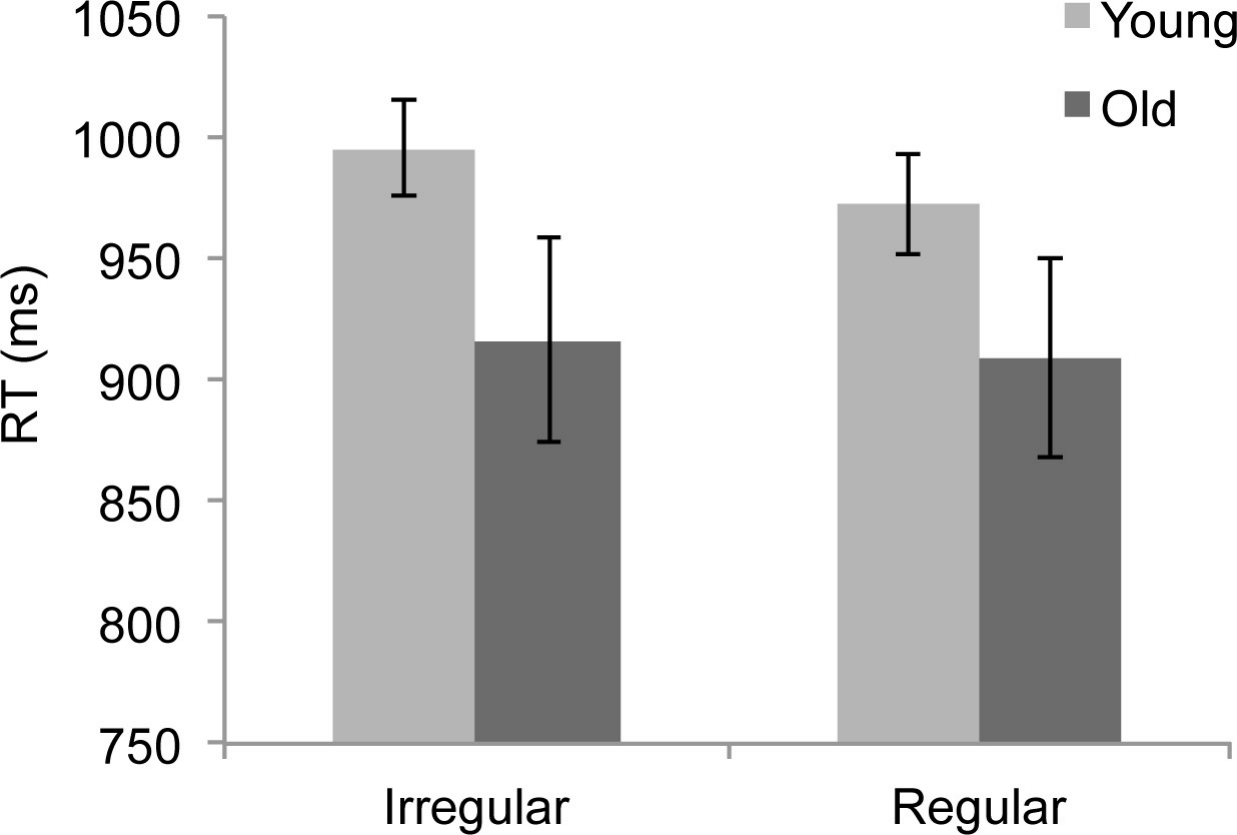
Response times. Mean RT data are shown for both groups of participants for the irregular and regular condition. Error bars reflect the SEM.

To evaluate the effects of aging and regularity on behavioral performance, we conducted an ANOVA for both the accuracy and response time (RT) data with a within-subjects factor of condition (2 levels: IC vs. RC) and a between subjects factor of group (2 levels: young vs. old). For the accuracy, we observed a main effect of condition (*F*(1,24) = 7.82, *p* = 0.01, η^2^ = .25), for which participants were more accurate in the RC (*M* = 94.3% correct) than the IC (*M* = 90.8% correct). The main effect of group was not significant (*p* = 0.5) and the interaction between group and condition also did not reach significance (*p* = 0.4). The RTs also showed a main effect of condition (*F*(1,24) = 7.45, *p* = 0.01, η^2^ = .24, with participants responding more quickly to the RC (*M* = 941 ms) than the IC (*M* = 956 ms). The RT data are shown in Fig 2. As with the accuracy, there was no main effect of group present in the RT data (*p* = 0.14), nor was there a group by condition interaction (*p* = 0.17). The accuracy and RTs were therefore influenced by the regularity of the stimuli, but this influence did not differ as a function of age.

### Awareness of learned patterns

To determine if any of the participants were aware of the patterns in the stimuli, we asked participants about what they noticed during the experiment. According to the self-reports, all elderly subjects were unaware of the underlying regularity in the regular condition. Two of the young subjects reported a “feeling” of some kind of regularity sometimes during the experiment. They were, however, unable to tell if this “feeling of regularity” happened more than one time, and could not concretely describe it.

### EEG data

**Fig 3:**
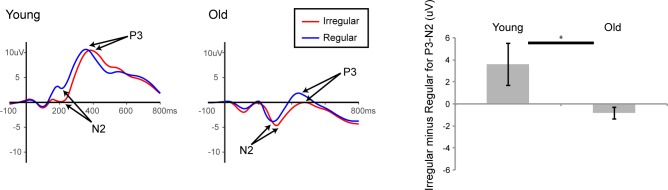
P_3_-N_2_ complex. The traces displayed on the left indicate the waveforms for the young participants’ irregular and regular conditions. The same traces for the older participants are shown in the middle of the figure. The bar graph on the right shows the mean amplitude for the P_3_-N_2_ complex for the irregular minus regular condition. The younger subjects differed from the older subjects in this pattern, as they showed a difference between the irregular and regular conditions, whereas the same pattern was not observed in the older subjects. The error bars reflect the SEM.

To analyze the overall effect of age and regularity, the P_3_-N_2_ amplitudes for the targets in the regular and the irregular condition were obtained for both the young and old subjects at site Pz. Then, separately, for each group, the difference between these amplitudes (irregular minus regular) was obtained (see Fig 3 for the waveforms and a graph of the differences). If regularity were detected, then one would expect to see a difference in these amplitudes between the irregular and regular condition. To determine if the groups differed in their detection of this regularity, we conducted a t-test for these amplitude differences between older and younger subjects. This revealed that the groups indeed significantly differed (*t*(24) = 2.22, *p* = 0.036), with younger adults showing the expected pattern for the detection of the regularity, and the older adults lacking this pattern.

**Fig 4:**
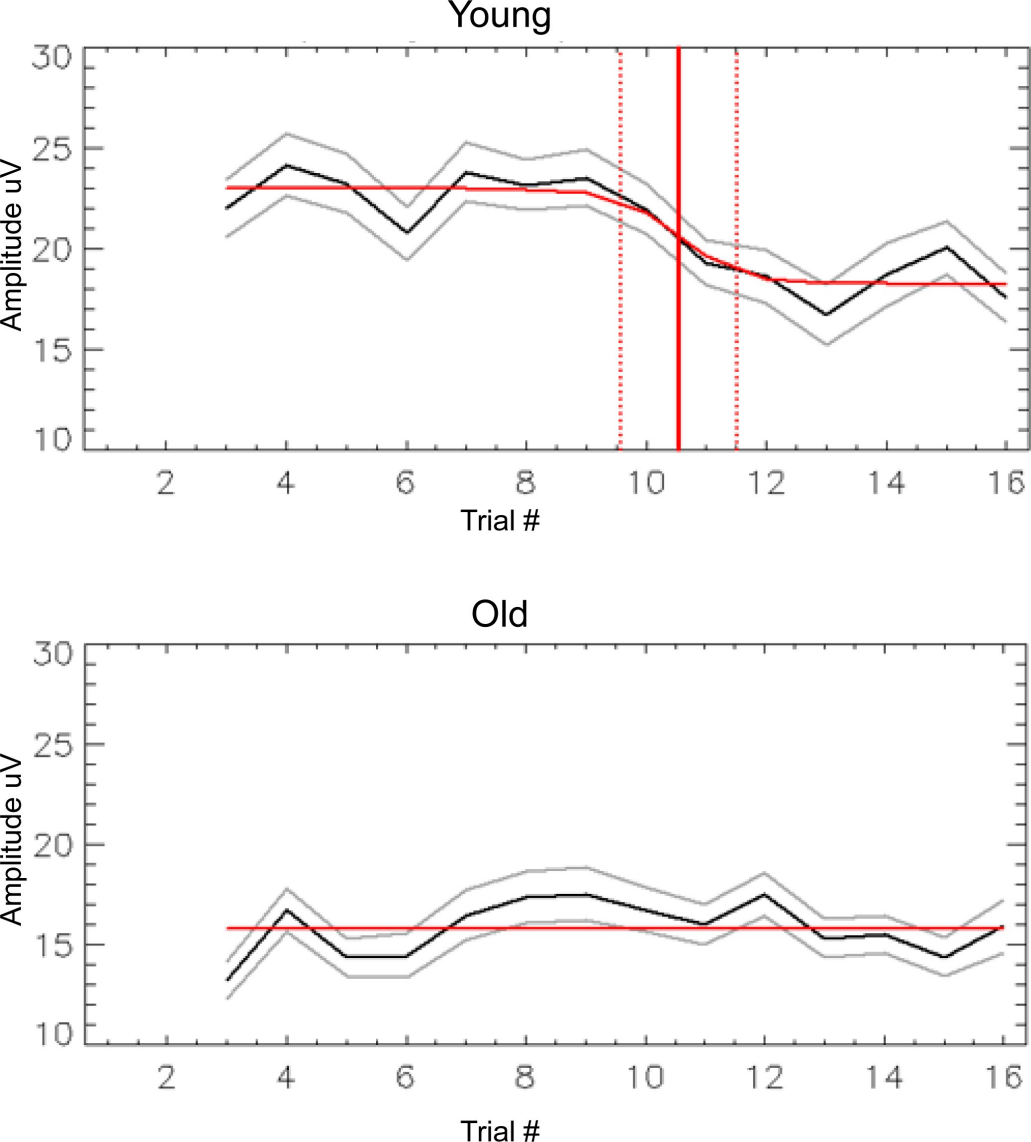
Sigmoid fits of data. Data averaged across cycles are depicted separately for the old group and the young group. At trial #9, the regular targets began being presented, and the young group shows a decrease in the amplitude of the P_3_-N_2_ complex subsequently. The old group showed no such decrease, and their data could not be significantly explained by a sigmoid function. The red vertical line represents the 50% value in the sigmoid curve (shortly after trial 10), and the red horizontal lines are the fitted data. The grey lines represent the 95% confidence intervals of the fit.

To determine the learning effect of the RC, we analyzed the P_3_-N_2_ complex of the single trials across each cycle and fit them with a sigmoid function (see Methods for details). Fig 4 depicts the fit of the data averaged across the cycles. For the young group, there was a significant effect of learning (*p* = 0.014), as well as a significant fit of the average of the cycles (*p* = 0.0001), whereas no such effects were present for the older group. The 50% mark of the sigmoid fit (i.e., 50% of amplitude reduction) for the young subjects occurred shortly after trial 10, suggesting that only two trials in the regular condition were needed before a decrease in the P_3_-N_2_ complex occurred.

## Discussion

In this study, we used EEG to examine the effects of aging on implicit pattern learning with an auditory paradigm and a single-trial denoising analysis technique developed by Jongsma and colleagues [25,32]. Two primary results emerged from these data. First, older participants did not differ from younger in their accuracy and response time in the task. Second, the neural signature of learning of regularity, the P_3_-N_2_ complex, did show a different pattern of activity in the older and younger subjects, with younger subjects showing differences in this complex across the regular and irregular conditions, and older subjects not showing a difference. Together, these data suggest that implicit auditory learning in younger and older subjects has different underlying neural mechanisms, but manifests similarly behaviorally.

The lack of a behavioral difference between the groups could be due to several factors. In this particular task, rather than having participants respond as quickly and as rapidly as possible, participants were instructed to withhold their response until they heard the non-target tone (which occurred 800 ms after the target tone). This delay may have provided the older adults enough time to prepare and execute a response, which is probably why they did not show the general slowing effects of response time often observed in tasks where an immediate response is required [33]. For both groups, we saw an overall increase in accuracy and decrease in response time for the RC compared to the IC, suggesting that both older and younger subjects had implicitly learned the regularity, even though no participant could concretely report said regularity. Although it is often the case that older and younger participants differ in performance on implicit learning tasks (e.g., [20]), similar performance across older and younger subjects has been observed under certain circumstances, particularly in cases where the to-be-learned pattern is relatively simple [9,10]. It is possible that the current task was fairly easy for participants because it was an auditory task, and the auditory modality has a natural propensity to detect temporal patterns [34]. Dennis and colleagues [12] also found (mostly) similar patterns of learning in younger and older adults in an auditory task, suggesting that perhaps implicit learning in aging is somewhat modality dependent, and spared more in the case of the auditory modality.

In the data fit across blocks, as well as in the data averaged across all trials, we were able to see differences between subjects in the P_3_-N_2_ complex, with younger subjects showing a decrease in this, where learning appears to be present after just three trials in the regular condition. Because both younger and older adults showed behavioral benefits for the RC compared to the IC, it is likely that the neural substrates of such implicit learning change in healthy aging. In the hypothesis put forth by Howard et al., [2] which has been supported by fMRI data [22,23], the striatum is not involved in implicit learning in older adults, but it is in younger adults. More specifically, Simon and colleagues [22] observed that as sequences become implicitly learned, younger adults shift from using the hippocampus to using the striatum for this, while older adults use only the hippocampus. Given that the hippocampus has been found to be one likely contributing source for the generation of the P_3_-N_2_ complex [35], if older adults were continuously using this structure throughout the task and younger adults shifted to using the striatum in the case of the learned patterns, then one would expect to find the difference between the RC and IC in the young adults and not in the older, as our results indicate.

These data provide further evidence for a neural compensatory mechanism that occurs in healthy aging (e.g., [36,37]). Despite the differences in brain activity, older adults did not show a behavioral deficit, suggesting a highly functional compensatory mechanism. Dennis and Cabeza [23] also found no behavioral differences between old and young subjects in implicit memory task performance, despite the recruitment of different neural structures. Moreover, given the lack of a difference in accuracy, it is likely that there were not global differences between the groups in terms of effort or attention. Finally, it should also be noted that none of our participants could report a pattern suggesting that any learning that did occur was entirely implicit and not relying on explicit knowledge, which is known to have a different pattern of neural activity in EEG [30].

To summarize, the present findings suggest that although older adults may still show some signs of implicit learning behaviorally, the neural hallmarks of pattern detection as observed in younger adults, are not present in older adults. It is likely that, as has been put forth (e.g., [2]), older adults are relying more on the MTL to compensate for the lack of striatal involvement in implicit learning tasks, and in the EEG data, this ramifies as a P_3_-N_2_ complex not differing across regularity conditions.
